# Quantifying the chemical activity of cavitation bubbles in a cluster

**DOI:** 10.1038/s41598-024-56906-5

**Published:** 2024-04-04

**Authors:** Kobra Fattahi, Daria C. Boffito, Etienne Robert

**Affiliations:** 1 Department of Chemical Engineering, Polytechnique Montréal, C.P. 6079, Succ. CV, Montreal, QC H3C 3A7 Canada; 2 Department of Mechanical Engineering, Polytechnique Montréal, C.P. 6079, Succ. CV, Montreal, QC H3C 3A7 Canada

**Keywords:** Engineering, Chemical engineering

## Abstract

Acoustic cavitation bubbles drive chemical processes through their dynamic lifecycle in liquids. These bubbles are abundant within sonoreactors, where their behavior becomes complex within clusters. This study quantifies their chemical effects within well-defined clusters using a new laser-based method. We focus a laser beam into water, inducing a breakdown that generates a single cavitation bubble. This bubble undergoes multiple collapses, releasing several shockwaves. These shockwaves propagate into the surrounding medium, leading to the formation of secondary bubbles near a reflector, separated from the input laser beam. We evaluate the chemical activity of these bubble clusters of various sizes by KI dosimetry, and to gain insights into their dynamics, we employ high-speed imaging. Hydrophone measurements show that conversion from focused shockwave energy to chemical reactions increases to a maximum of 16.5%. Additional increases in shockwave energy result in denser bubble clusters and a slightly decreased conversion rate, falling to 14.9%, highlighting the key role of bubble dynamics in the transformation of mechanical to chemical energy and as a result in the efficiency of the sonoreactors. The size and frequency of bubble collapses influence the cluster’s chemical reactivity. We introduce a correlation for predicting the conversion rate of cluster energy to chemical energy, based on the cluster’s energy density. The maximum conversion rate occurs at a cluster energy density of 2500 J/L, linked to a cluster with an average bubble diameter of 91 $\upmu$m, a bubble density of 3500 bubbles/ml, and a bubble-to-bubble distance ratio of 8.

## Introduction

Cavitation bubbles can form in liquids when the local pressure drops below the saturated vapor pressure. Traditionally, cavitation bubbles have been considered problematic in hydraulic systems due to their ability to cause erosion on surfaces and generate noise as a consequence of their violent collapse that yields liquid jets, high temperatures and pressures^1^. However, the same concentration of energy that causes these problems can have useful applications in various fields, such as wastewater treatment^2^, surface cleaning^3^, and chemical synthesis (in heterogeneous and homogeneous systems)^4–8^. Acoustically-induced cavitation bubbles are at the forefront of these applications, as a large bubble number density within the treated medium leads to intense sonochemical reactions. Sonochemistry involves the study and application of chemical reactions and processes that are influenced or enhanced by acoustically-induced cavitation bubbles. During this process, the bubbles experience oscillating pressure waves that cause them to grow and collapse violently, generating temperatures and pressures high enough to initiate chemical reactions that would not occur under normal conditions^9^.

**Figure 1:**
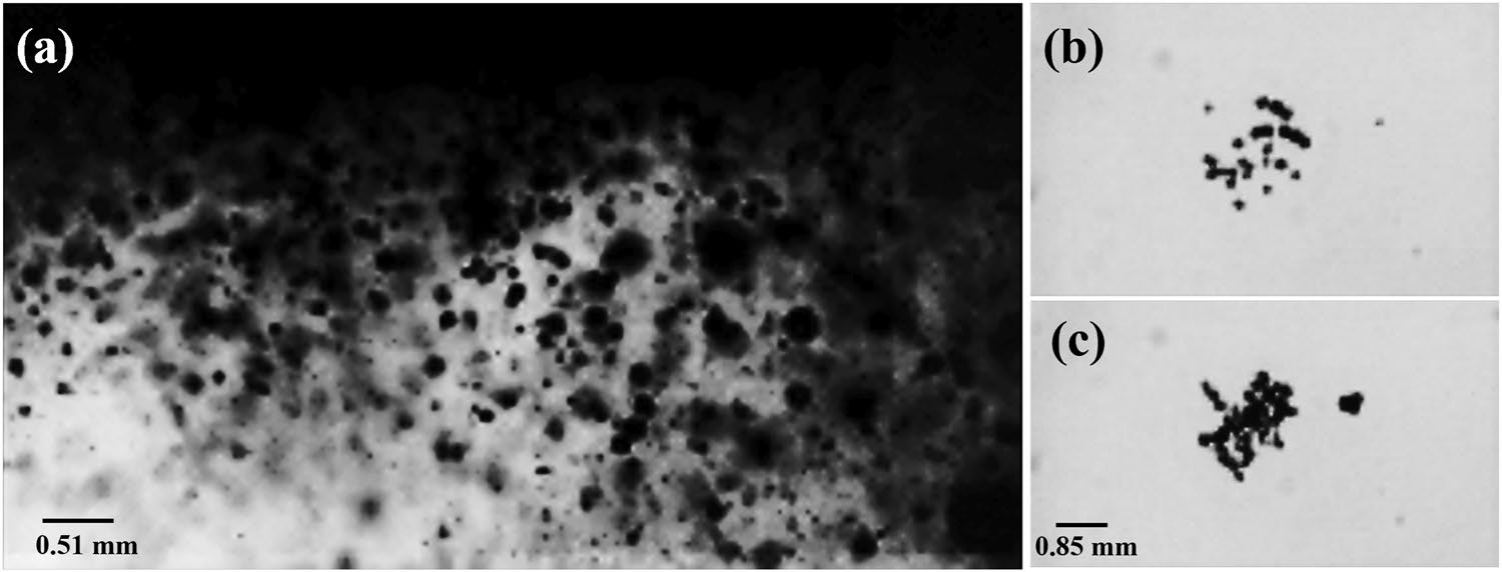
Cavitation bubbles formed by (**a**) a 20 kHz horn-type transducer, subjected to an ultrasound power of 70.43 W, captured near the tip of the probe using high-speed recording at 100k fps, (**b**) laser breakdown shockwaves (BSW), and (**c**) laser-induced bubble collapse shockwave (CSW), high-speed recordings at 60k fps.

Acoustic cavitation bubbles occur in large numbers and complex patterns, including clusters and clouds of bubbles within cavitation zones of sonoreactors and cleaning baths^10^ (Fig. 1a). Compared to isolated single bubbles, cavitation bubbles in clusters exhibit different behavior due to bubble-bubble interactions and coalescence, which makes the mechanism that promotes chemical activity still unclear. Although considerable progress has been made in our understanding of the dynamics of single bubbles^11^, our comprehension of bubble cluster dynamics and their chemical activity in a sound field is still limited^12^. The study of a well-defined bubble cluster presents significant challenges and has been the focus of numerous research efforts^13–16^. Despite these efforts, there remains a gap in our understanding of the relationship between bubble clusters dynamics and their chemical activity.

To achieve a comprehensive understanding of the behavior and chemical activity of cavitation bubbles in sonoreactors, it is crucial to gain fundamental insights into the behavior of bubbles within clusters. Thus, the availability of experiments that enable the investigation of cavitation bubble clusters in well-defined conditions (Fig. 1b, c), including the influence of bubble parameters on the chemical activity within the cluster, is key to design efficient and effective sonochemical processes.

Cavitation bubbles can be generated in experiments using various methods, including explosions^17^, spark discharge^18^, ultrasonic waves^19^, and laser pulses^20^. While detonation of explosive charges leads to the formation of large bubbles with extended lifetime, bubble inception time is challenging to control, and safety concerns must be thoroughly evaluated. Additionally, the explosive determines the gas content in the bubble, which affects the chemical reactions in the medium^17,21,22^. The use of spark-induced bubble formation has limitations due to the impact of the electrode immersed in the liquid medium on bubble dynamics^18,23^. Among the various methods available, ultrasound-induced cavitation bubbles are widely utilized in enhanced chemistry studies due to their dynamic behavior and high yield of sonochemical reactions^1,8,16,19,24–27^. Different types of transducers, including horn^19^, bath^28^, and focused transducers^29^, are used to generate ultrasound waves and cavitation bubbles. However, achieving precise control over the inception time, number density, and position of the bubbles for studying their behavior is challenging with horn and bath-type transducers due to various influencing factors, such as spatial positioning, interactions with nearby clusters, acoustic streaming, and reactor conditions^19,30^. Even with focused transducers, the movement of the transducer surface combined with the propagation of sound waves in the liquid medium generate streaming, which can impact the dynamics of the bubbles and potentially interfere with the desired experimental conditions^30^. Therefore, to gain a comprehensive understanding of the chemical activity of bubble clusters under controlled conditions, we utilized laser pulses to generate cavitation bubbles. This method offers easy control over the creation time and inception point of the laser-induced bubble and is considered a non-invasive, precise, and highly localized approach that produces very symmetrical bubbles^17,18,31^. However, with this technique, generally, only a single bubble is generated in the medium, and thermal effects in the plasma induced by the focused laser beam affect the chemical reaction.

Consequently, we introduce modifications to the laser-induced cavitation approach to ensure its suitability for our study. We focus a pulsed laser beam into a water medium to provide the energy necessary to form a single cavitation bubble. When the optical irradiance in the focal region reaches a critical value known as the breakdown threshold of water ($I_{Th} = 8 \times 10^9 \, \text {W/cm}^2$)^32^, a small volume of liquid heats up to ionization temperatures and forms plasma, leading to explosive expansion and the formation of a cavitation bubble. The laser-induced bubble is not in equilibrium with the surrounding liquid and expands until it reaches its maximum radius before beginning to recede until it collapses^33–35^.

Two primary processes result in the formation of reactive species in laser-induced cavitation bubbles. The first is the ionization and dissociation of water in the focal volume of the laser beam, producing a dense plasma containing hydrated electrons ($\hbox {e}_{\textrm{aq}}^{-}$), hydrogen ions ($\hbox {H}_{3}\hbox {O}^{+}$), hydroxyl radicals (${\hbox {OH}^{.}}$) and hydrogen atoms. At the end of the exciting pulse, recombination reactions occur during plasma expansion and cooling, transforming the produced ions, radicals, and atoms into water ($\hbox {H}_{2}\hbox {O}$), hydrogen peroxide ($\hbox {H}_{2}\hbox {O}_{2}$), oxygen ($\hbox {O}_{2}$), and hydrogen ($\hbox {H}_{2}$)^31,36^. The second process is associated with the violent collapse of the bubble. Nanosecond laser pulses containing a few mJ energy can create a millimeter-sized bubble, which is then compressed under ambient liquid conditions, leading to temperatures of over 7000 K and pressures of several thousand bars within the collapsing bubble^1,37^. These extreme conditions give rise to the formation of new reactive species, a phenomenon also observed in sonochemistry. One such species is hydroxyl radicals, which are generated and diffuse into the surrounding liquid, actively participating in various chemical reactions^31^.

The collapse of laser-induced cavitation bubbles in water generates multiple shockwaves, which can be visualized as spheres expanding radially from the laser’s focal point in optically transparent liquids^38^. The first shockwave (breakdown shockwave-BSW) detaches from the plasma and leaves behind an oscillatory cavitation bubble, whose liquid-vapor boundary expands and then contracts until it collapses due to the pressure of the surrounding liquid and releases another shockwave (bubble collapse shockwave-CSW). During the second oscillation, the cavitation bubble expands to a smaller maximum radius than the first oscillation (as energy is lost from the first shockwave) before imploding again and releasing another CSW, and so on^39^. By employing a concave boundary, we are able to intercept and focus these initially divergent shockwaves, which allows the formation of secondary cavitation bubbles within a separate liquid medium that is isolated from the laser pulse. Our use of the laser pulse technique presents a novel approach to investigate the dynamics and chemical activity of both individual laser-induced bubbles and clusters of secondary cavitation bubbles. This approach eliminates the confounding effects of the laser pulse itself, enabling a focused exploration of the chemical effects associated with the bubble cluster phenomenon.

This research introduces this novel method of generating cavitation bubble clusters in water using laser pulses and quantifies experimentally the number density and size of these bubbles, as well as the oxidative chemical reactions that take place within the laser-induced bubble and the secondary cavitation bubbles medium. Despite numerous studies having been conducted on laser-induced bubbles and cavitation bubbles in water, this work is significant as it represents the first attempt to quantify the chemical activity of the bubble clusters formed by the reflection of shockwaves in a liquid medium without perturbing influences, such as strong acoustic streaming or the presence of nearby walls or dense bubble clouds preventing the visualization of bubble dynamics. To quantitatively analyze the chemical activity, we use the KI dosimetry technique. The laser pulse energy and irradiation duration time are the main adjustable parameters, and we measure their impact on the produced reactive species. The empirical correlation derived from our experimental results on secondary bubbles provides a predictive model for the conversion of source acoustical energy into chemical energy through the formation of a bubble cluster with a known distribution of bubble number density and size. The way we control the energy and behavior of the bubbles plays a major role in how efficiently their potential energy can be converted into chemical activity. This understanding can help in the design of more effective ultrasound-based techniques.

## Material and methods

### Experimental setup

**Figure 2:**
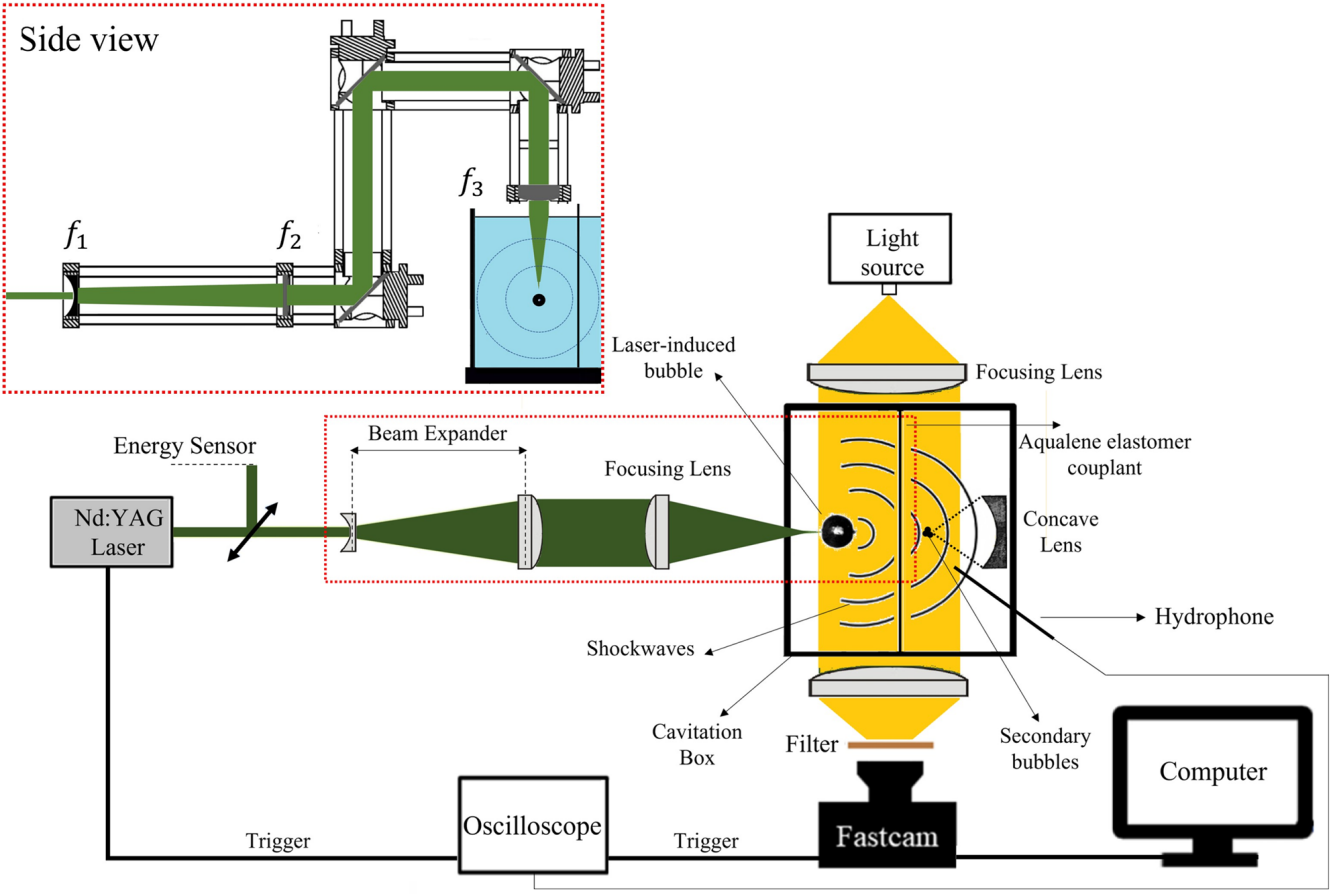
Experimental setup for the high-speed imaging of laser-induced and secondary cavitation bubble formation.

The experiments are conducted at ambient pressure. Before the experiment, we degas distilled water for 10 minutes on both sides of the cavitation box to reduce the formation of small bubbles after the collapse of the main ones, which facilitates the propagation of shockwaves in the liquid medium. As depicted in Fig. 2, the experimental setup can be broadly divided into three main parts: (1) a bubble nucleation apparatus, (2) a cavitation box, and (3) a video recording system.

#### Bubble nucleation

Bubbles are generated by focusing a pulsed laser into a cavitation box filled with pure distilled water. The laser is a frequency-doubled Q-switched Nd:YAG (Litron Nano S 35-15) that emits light pulses at a wavelength of 532 nm and with a duration of 4 ns. The laser repetition rate can be adjusted between 0 and 10 Hz. The laser energy, $E_{T}$, at the exit is measured by an energy sensor through a beam splitter. For the experiments presented here, the laser energy is varied between 55 and 95 mJ. The repetition frequency is set at 10 Hz, and the irradiation time is between 10 and 20 minutes, for a total of 6000–12000 laser pulses per experiment. To ensure a compact plasma and prevent multiple breakdown sites and bubbles with poor sphericity, the laser beam is expanded before focusing. This is achieved through a custom-made telescope system consisting of a plano-concave spherical lens ($f_{1}$= $-$ 50 mm) and a plano-convex spherical lens ($f_{2}$ = 125 mm), creating a $\times 2.5$ expansion of the original laser beam (75 mm after expansion). The expanded beam is focused in the volume of water from the top of the cavitation box by using three dielectric-coated mirrors and a plano-convex spherical lens ($f_{3}$ = 35 mm) (as seen in the side view of Fig. 2).

#### Cavitation box

The expanded and then focused laser beam is introduced into a quartz cavitation box ($7 \times 7 \times 2.5\,\hbox {cm}^{3}$). The box is divided into two sections using an aqualene elastomer couplant of 0.5 mm thickness (29HD0010, OLYMPUS, USA). This material has an acoustic impedance close to that of water ($1.49 \times 10^6\,\hbox {kg/m}^{2}\hbox {s}$) and a low attenuation coefficient, making it ideal to transmit the shockwaves between the two sides of the cavitation box. The laser beam is focused in one side of the box through the free surface at the top, creating the laser-induced bubble. A parabolic lens (Thorlabs, effective focal length 9 mm, radius of curvature 7 mm) is placed on the opposite side of the cavitation box to reflect the shockwaves and generate secondary cavitation bubbles. A precision translation stage (Thorlabs, DTS.50) is used to accurately position the reflector relative to the laser focal point and keep it in the same place for all experiments.

#### Bubble imaging

An oscilloscope is used to synchronize the bubble imaging system from the output signal of the laser. The imaging system consists of a flicker-free fiber optic lamp (Thorlabs OSL2, Inc) modified with plano-convex lenses (150 mm focal length and 50 mm diameter) to create a collimated 2-inch diameter illuminated field. We capture the behavior of laser-induced and secondary cavitation bubbles in optically transparent media using a high-speed video camera (FASTCAM Ax200, Photron, Japan) equipped with a Micro-Nikon 105 mm f/2.8G lens. To prevent saturation and damage to the camera sensor, a long-pass filter is placed in front of the lens to suppress the green pulsed laser at 532 nm (see Fig. 2). Videos of the laser-induced bubble are recorded at 37.5 k fps and an exposure time of 1/300000 s, while videos of secondary bubbles are recorded at 60k fps. The cavitation bubbles appear as black shadows in the images, with a spatial resolution of 29 $\upmu$m/pixel.

### Pressure measurements

Hydrophone measurements are used to detect the shockwaves generated by the laser-induced breakdown and to determine the energy of the shockwaves that passed through the aqualene sheet and emerged on the other side of the cavitation box. The bubble period, defined as the time interval between successive cycles of a cavitation bubble, is precisely measured by detecting the shockwaves emitted during the process of bubble generation and subsequent collapse. For a spherical bubble, the time delay between the two shockwaves emitted is equal to twice the collapse time of the bubble^32^.

For these purposes, we employ a PVDF hydrophone, which is a piezoelectric device that converts underwater sound pressure into electrical signals to measure the shockwave profile^40^. We utilize a manual positioning system to accurately locate the hydrophone (Muller-PVDF, needle-type with a rise time of 50 ns) during field mapping. Employing only one hydrophone, we carefully change its position from the laser-induced bubble side to the secondary bubbles side. In both locations, the hydrophone is positioned at the same angle relative to the direction of shockwave propagation, ensuring consistent and comparable measurements. It is positioned with a standoff distance of approximately 8 mm from the laser-induced bubbles and 5 mm from the secondary bubbles, respectively, within the cavitation box. The hydrophone voltage is read directly on an oscilloscope (viewGo, Iwatsu Co., Japan).

Based on the findings of Vogel and Lauterborn^39,41^, it has been determined that the duration of shockwaves, regardless of their origin from either optical breakdown or cavitation bubble collapse, is shorter than 20 ns. Consequently, directly measuring the maximum pressure of these shockwaves from the hydrophone recordings becomes impossible. This limitation arises from the fact that the duration of the pressure pulse is shorter than the rise time of our hydrophone (50 ns), resulting in the hydrophone not capturing the complete pressure value. Thus in the immediate vicinity of the plasma, the parameter *P* has, so far, only been determined through numerical calculations^42^.

In the domain of linear sound propagation, the pressure pulse amplitude diminishes in inverse proportion to the distance (r)^39^. However, within the shock wave area, the pressure decline surpasses the 1/r rate, largely because sound energy strongly dissipates into heat at the shock front. As highlighted by Lai’s research^43^, the decrease in the 0.2 mm $\le$ r $\le$ 1 mm region has a slope greater than 1, roughly averaging to $r^{1.5}$. The energy of these spherical acoustic transients can be determined by^41,44^:

1 $$\begin{aligned} E_{C}= \frac{4 \pi r^2}{\rho c} \int P^2(t) \, dt \end{aligned}$$

where *c* is the speed of sound in water, 1450 m/s; $\rho$ is the density of water, $10^3\,\text {kg/m}^3$; *r* is the distance between the pressure transducer and the emission center of the acoustic transient; *P* is the shockwave pressure (Pa). In the far field, much of the shockwave energy dissipates, making measurements taken at a distance from the source inaccurate in representing the total acoustic energy generated during optical breakdown.

### KI method

To quantify the amount of reactive oxygen species (ROS), $\hbox {OH}^{.}$ radicals, we conduct KI dosimetry. The concentration of triiodide ions oxidized by $\hbox {OH}^{.}$ radicals is measured at 350 nm using a UV-vis spectrophotometer (Vibra S60, Biochrom Ltd., UK)^28^. The average diffusion length of $\hbox {OH}^{.}$ in the liquid phase is 240 nm^45^, and they exhibit an exceptionally brief lifetime (less than 10 ns in liquid) relative to their production rate^46^. Within this short $\hbox {OH}^{.}$ diffusion length and lifetime they can either interact with other gaseous species or recombine to generate hydrogen peroxide ($\hbox {H}_{2}\hbox {O}_{2}$), one of the long-lived species generated^46^. In this method, when potassium iodide (KI) solution is sonicated, oxidation occurs and $\hbox {I}^{-}$ ions are oxidized by the $\hbox {H}_{2}\hbox {O}_{2}$ to give $\hbox {I}_{2}$. The excess of $\hbox {I}^{-}$ ions present in the solution reacts with $\hbox {I}_{2}$ to form triiodide ion ($\hbox {I}_{3}^{-}$)^47^. The reaction mechanism is as follows:  ^48^

$$\hbox {H}_{2}\hbox {O} \rightarrow \hbox {OH}^{.} + \hbox {H}^{.}$$

$$2 \hbox {OH}^{.} \rightarrow \hbox {H}_{2}\hbox {O}_{2}$$

$$2 \hbox {H}^{.} \rightarrow \hbox {H}_{2}$$

$$\hbox {H}_{2}\hbox {O}_{2} + 2 \hbox {I}^{-} + 2 \hbox {H}^{+} \rightarrow 2 \hbox {H}_{2}\hbox {O} + \hbox {I}_{2}$$

$$\hbox {I}_{2} + \hbox {I}^{-} \rightarrow \hbox {I}_{3}^{-}$$

The KI solution (Sigma-Aldrich Co., St. Louis, Mo, USA) is prepared with a concentration of 0.9 mol/L. Each experimental test is independently conducted, commencing from zero minutes. Following the end of each test at predefined intervals of 10, 15, and 20 minutes, the solution is thoroughly mixed and 25 ml of the solution is sampled using a syringe. 2 ml of the ejected solution is then placed into a quartz cell with a 1 cm optical path length for analysis. All measurements are performed two times, and the average values are reported in this study.

To establish a relationship between the dynamics of the bubbles in a cluster (size and number density) and chemical activity, the number of moles of $\hbox {I}_{3}^{-}$ is determined based on the UV light absorbance of the solution using the Beer-Lambert law. In this measurement, the UV light absorbance of the solution before the laser pulse is subtracted from the absorbance after exposure to the laser pulses^49^:

2 $$\begin{aligned} A=C\times \varepsilon \times l \end{aligned}$$

where A is UV light absorbance; C is the concentration of triiodide ion (mol/L), $\varepsilon$ is the extinction coefficient of $\hbox {I}_{3}^{-}$ (26,303 L/mol cm), and l is the cuvette length (l = 1 cm).

Considering the reaction between the $\hbox {I}^{-}$ ion and $\hbox {H}_{2}\hbox {O}_{2}$ in an aqueous solution, the mole quantity of $\hbox {H}_{2}\hbox {O}_{2}$ is equivalent to that of $\hbox {I}_{3}^{-}$ (overall stoichiometry: $\hbox {H}_{2}\hbox {O}_{2} + 3 \hbox {I}^{-} + 2 \hbox {H}^{+}\rightarrow \hbox {I}_{3}^{-} + 2 \hbox {H}_{2}\hbox {O}$). Furthermore, through the recombination reaction of hydroxyl radicals, the number of $\hbox {OH}^{.}$ moles can be determined by doubling the moles of $\hbox {H}_{2}\hbox {O}_{2}$.

## Results and discussion

### Shockwave signal

**Figure 3:**
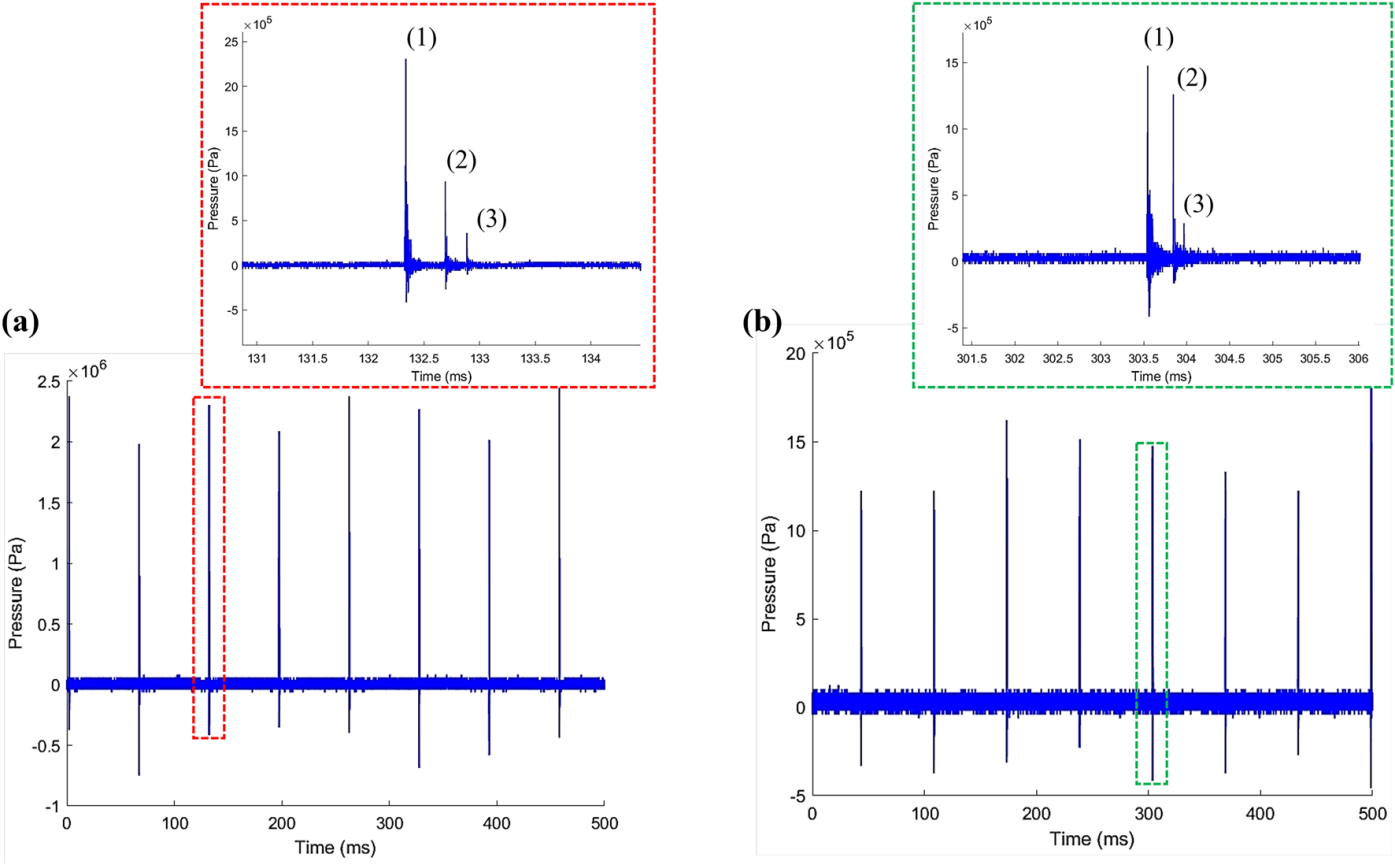
Temporal pressure profile captured by hydrophone subjected to laser energy of 81 mJ: (1) shockwave emitted by the laser-induced breakdown. (2) and (3) shockwaves emitted by the first and second rebound of the laser-induced bubble, respectively.

We observe that shockwaves generated by bubbles formed by laser energy below 55 mJ do not result in the formation of any secondary cavitation bubbles. As a result, we established a minimum laser energy threshold of 55 mJ for our experiments. At each laser energy, multiple shockwaves are recorded by the hydrophone due to plasma expansion and subsequent cavitation bubbles formation (Fig. 3). Shockwaves are detected on both sides of the cavitation box. We identify three peaks labeled as peaks 1, 2, and 3 by zooming in on the signal detected following each laser pulse on both sides of the cavitation box. The first peak corresponds to the bubble’s expansion (BSW), while the second and third peaks correspond to the first and second collapses of the bubble (CSWs), respectively (Fig. 3)^32,50^.

**Figure 4:**
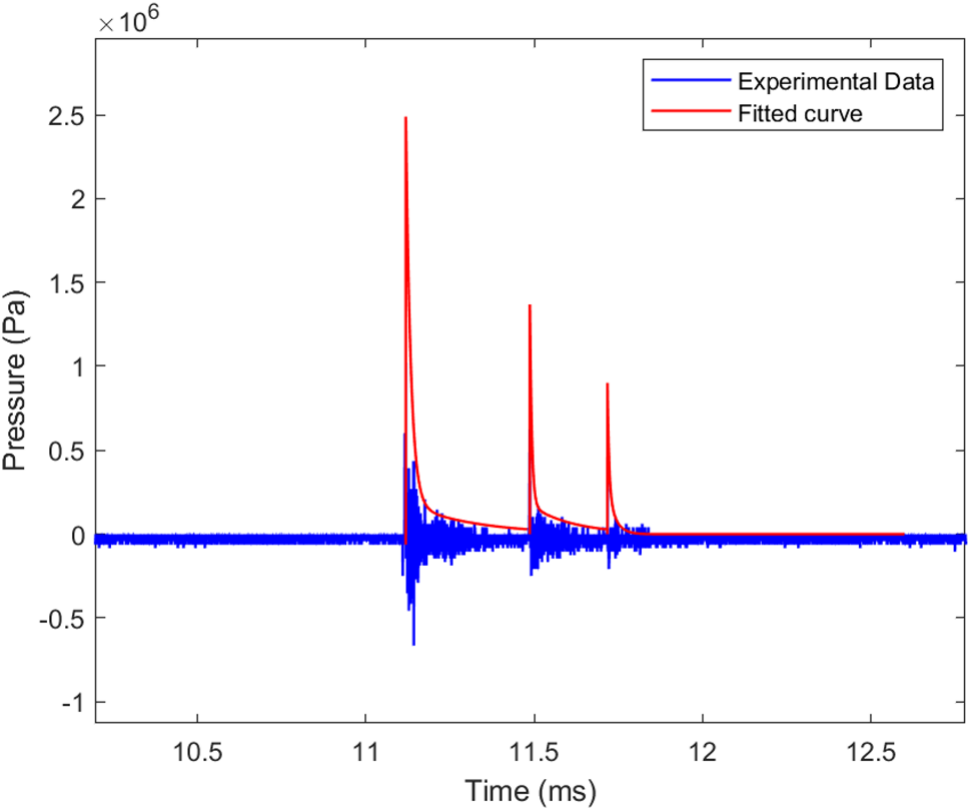
Typical pressure versus time graph and the decayed curve fitted on the experimental data from a single 81 mJ laser pulse, measured 8 mm from the source.

Figure 4 illustrates the shockwaves produced during a single laser pulse event on the laser-induced bubble side of the cavitation box, and at a distance of 8 mm from the focal point. In this instance, the shockwave displays a characteristic shape featuring a steep shock front and an exponentially decaying tail. The incident shockwave arrives with a peak value of 2.5 MPa and rapidly decays thereafter. Following the initial pulse, two subsequent shockwaves are observed, displaying similar profiles but with lower maximum pressures.

**Figure 5:**
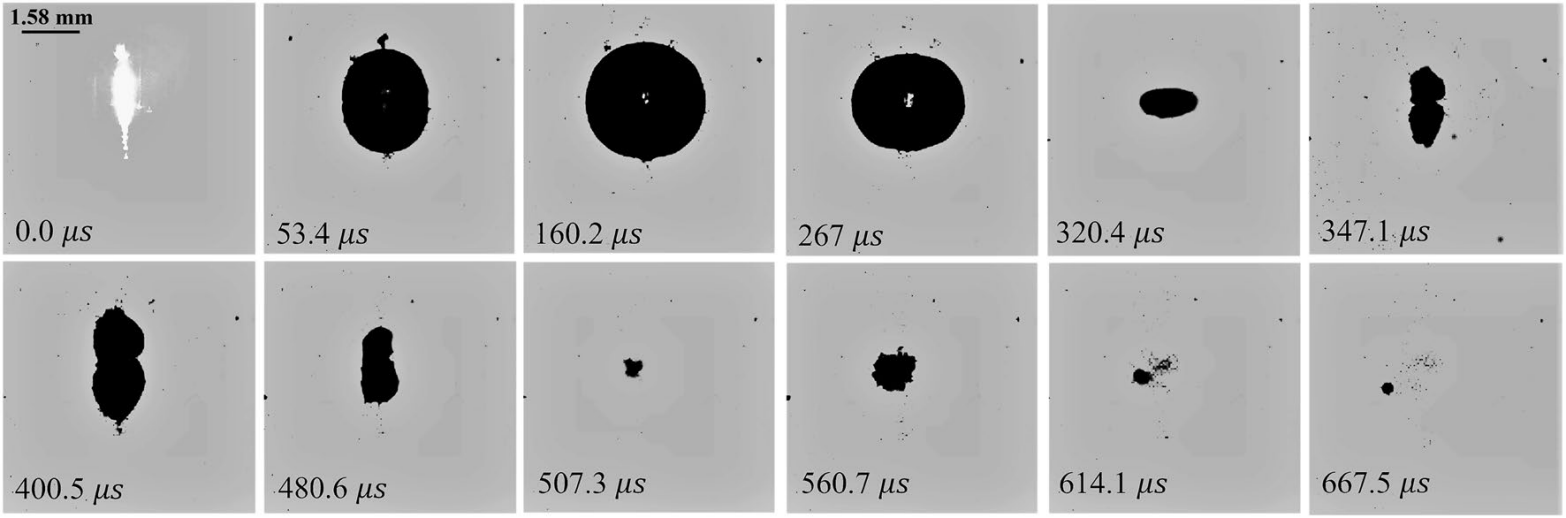
High-speed camera images (37.5K fps) of a laser-induced bubble at 55 mJ laser pulse energy.

Figure 5 demonstrates that in our system, laser-induced bubbles typically undergo three collapses. However, the hydrophone signal (see Figs. 3 and 4) consistently detects shockwaves generated only from the first two collapses. While the third collapse occurs, it does not reliably lead to the formation of a detectable shockwave by the hydrophone, indicating a likely decrease in the intensity and impact of this final collapse. Typically, following the first collapse of the bubble, disturbances in its sphericity occur, leading to an irregular second collapse. This is then followed by a significant attenuation of the bubble’s oscillation and its rapid decay into microbubbles^39^. Hence, shockwaves are reliably detected only after the breakdown, the first and second bubble collapse.

The absorbed laser energy is divided into three pathways: evaporation, plasma radiation and mechanical effects such as shock wave generation, and cavitation^51^. The descriptions of all terms used in this study are summarized in Table 1 to enhance understanding and readability.

**Table 1:** Summary of energy levels and their descriptions.

Energy variable	Description
$E_T$	Total laser energy delivered into the water medium
$E_{s1}$	Initial shockwave energy generated by water breakdown
$E_{s2}$	Shockwave energy generated by first bubble collapse
$E_{s3}$	Shockwave energy generated by second bubble collapse
$E_S$	Cumulative energy transferred through all generated shockwaves
$E_B$	Stored energy within the laser-induced bubble at its maximum potential state
$E_{r1}$	Chemical reaction energy on the laser side within the cavitation box
$E_{r2}$	Chemical reaction energy on the cluster side within the cavitation box
$E_b$	Energy retained within the secondary cavitation bubbles
$E_{SS}$	Energy component of the shockwave reflected by the parabolic lens

Measuring the energy of the cavitation bubble ($E_B$) is relatively straightforward, which is discussed in Sect. “Size and number of cavitation bubbles”. However, determining the shock wave energy ($E_S$) is more challenging. The energy carried away by these strong shockwaves, both during water breakdown and each cavitation bubble collapse, can be estimated using the hydrophone signal. The pressure pulse amplitude is derived from the pressure profile at $r=8$ mm. Using the $1/r^{1.5}$ law, we calculate the peak pressure 200 $\upmu$m away from the source. Lai et al.^43^ reported that the duration of a shockwave, defined as the time it maintains a pressure higher than the surrounding pressure, is approximately 50 ns at $r\approx 200 \upmu$m. The detailed results of the method used to determine the shock wave energy are presented in Table 2. The values listed in the table are the averaged values over 10 laser pulses. It presents the relationship between the laser energy ($E_{T}$) and the energy of the first ($E_{s1}$), second ($E_{s2}$), and third shockwaves ($E_{s3}$), as well as the ratio of the total shockwave energy ($E_{S}$) to the input laser pulse energy.

**Table 2:** Summary of results for the shock wave emission and the energy partition at $r=0.2$ mm.

$E_T$ (mJ)	55	74	81	95
$E_{s1}$ (mJ)	1.9	2.7	2.8	3.9
$E_{s2}$ (mJ)	0.4	0.5	0.6	1.1
$E_{s3}$ (mJ)	0.03	0.04	0.05	0.2
$E_{S}$/$E_T$ (%)	4.4	4.5	4.2	5.4

As the laser energy $E_T$ increases from 55 to 95 mJ, the energy of the first, second, and third shockwaves correspondingly increases. The energy ratios between the shockwave ($E_S$) and the laser ($E_T$) are quite low in our experiments. This shortfall can be attributed to laser beam quality issues, such as stability, potentially resulting in a decrease in the actual energy delivered to the focal region compared to its initial value. Consequently, the actual conversion rate may slightly exceed the observed 5.42% in our most favorable scenario. Nevertheless, this fact will not affect the results of the experiments, as what truly matters is the energy of the shockwaves at the reflector position and their contribution to the formation of the secondary cavitation bubbles.

### Distribution of cavitation bubbles

Figure 5 depicts the dynamics of the laser-induced bubble immediately after the laser pulse exposure (only selected frames are displayed). Using the focused direct shadowgraphy technique, the first $870\,{\upmu }\hbox {s}$ following the optical breakdown are captured and visualized with a time increment of $26.7\,{\upmu }\hbox {s}$ between each frame. These images clearly show the growth, collapse, and rebound of the laser-induced bubble.

The focusing angle of 10.13^∘^ used for the laser beam, combined with relatively large pulse energy, resulted in an approximately spherical laser-induced bubble. However, slight asymmetry is introduced into the cavitation bubble growth due to the elongated plasma, as seen in the first frame of Fig. 5. After the first collapse, the cavitation bubbles assume an asymmetric peanut-like shape, which is not observed after the second rebound.

**Figure 6:**
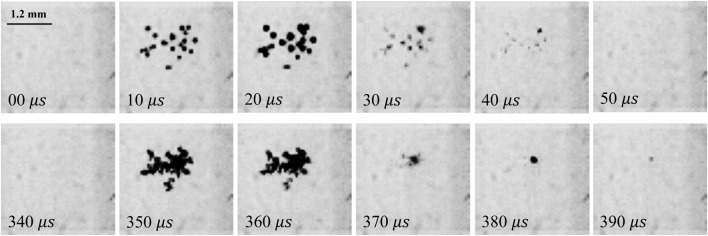
Time-lapse images of secondary bubble clusters during the passage of two shockwaves through the reflector’s focal point, captured with high-speed imaging at 100 k fps and a laser pulse energy of 74 mJ.

The same high-speed camera is employed to observe the inception of small cavitation bubbles near the focal point of the reflector (concave lens) on the other side of the cavitation box. Figure 6 shows these secondary bubbles soon after the reflected BSW and the CSW pass over the focus point of the reflector. In the early phase of the shockwaves’ spherical expansion, no cavitation bubbles are formed. However, upon reflection at the boundaries of a finite reflecting aperture, tensile “edge waves” are generated^38^. The acoustic impedance of the parabolic glass lens is calculated as $Z= 2230 \, \hbox {kg/m}^{3} \times 5640 \, \hbox {m/s}$, which is significantly greater than the acoustic impedance of water^52^. This substantial difference in acoustic impedance between water and the reflector ensures that the shockwave reflection occurs without pressure inversion. Consequently, the tensile stress arising from the converging edge waves surpasses the cavitation threshold, thereby inducing the formation of secondary cavitation bubbles^38^.

**Figure 7:**
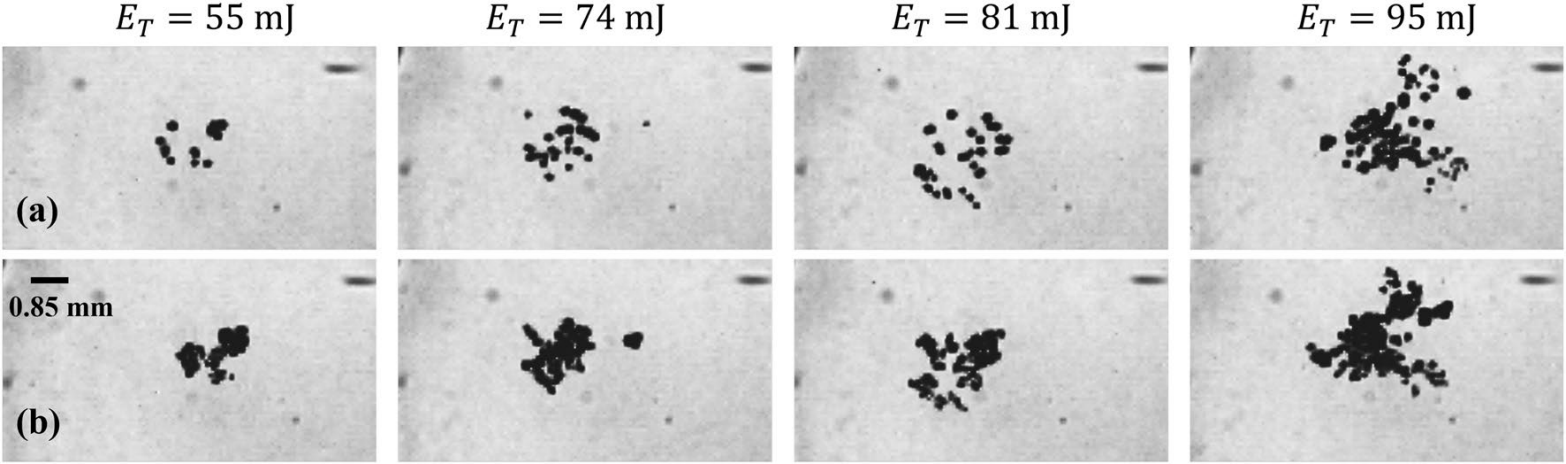
Images of the two sets of secondary bubble clusters: (**a**) first set, (**b**) second set, at their maximum size under different laser energies, captured using high-speed imaging at 60 k fps.

The secondary bubbles collapse and reappear with varying sizes, forming two sets of clusters at the focal point after each laser pulse (Fig. 6). The first set results from the BSW, while the second set is due to the first CSW of the laser-induced bubble. Although the size of the clusters may slightly differ between sequences, they consistently form twice per laser pulse near the reflector’s focal point. Figure 7 displays both sets of clusters when they each reach their maximum size under different laser energy levels and it is evident that the sizes of the clusters increase proportionally with the increase in laser energy.

Based on the hydrophone signal and the presence of three detected shockwaves on both sides of the cavitation box, it is anticipated to observe three sets of clusters per laser pulse. Furthermore, secondary bubbles also undergo collapses, generating additional shockwaves. However, these shockwaves resulting from the second bubble collapse and the secondary bubbles are insufficiently powerful to form tertiary cavitation when refocused.

### Size and number of cavitation bubbles

The high-speed camera records the dynamic behavior of a laser-induced bubble over a one-second duration, which corresponds to 10 laser pulses, as the laser pulse energy is varied from 55 to 95 mJ. The bubble’s size is determined by converting the recorded images into binary images using a Matlab code. Table 3 summarizes the results, including the average maximum radius of the bubble obtained from high-speed imaging and using the Rayleigh-Plesset model ($R_{c}$), relying on the hydrophone signal to obtain the duration of the bubble first oscillation (Eq. 3). The Rayleigh-Plesset model assumes spherical symmetry for the bubble throughout its lifetime and is derived under the assumption of an infinite and incompressible liquid, ignoring the influence of the surface tension and the dynamic viscosity of water^38^.

3 $$\begin{aligned} R_{c}= \frac{t_{c}}{\zeta \sqrt{\rho /(p_{\infty }-p_{v})}} \end{aligned}$$

Here $\zeta = 0.914681$ is the Rayleigh factor, $t_{c}$ is the collapse time, $\rho$ is the density of water, $p_{v}$ is the saturated vapor pressure, and $p_{\infty }$ is the constant far-field pressure equal to the ambient pressure.

The amount of energy available to form radicals can be estimated from the energy loss during bubble collapse, which is obtained by comparing the energy before and after a rebound. The potential energy $\hbox {E}_{\textrm{B}}$ contained in the cavitation bubble is given by^32^:

4 $$\begin{aligned} E_{B}= \frac{4}{3} \pi (p_{\infty }-p_{v}) R_{max}^3 \end{aligned}$$

Where $R_{max}$ is the maximum radius of the bubble.

Increasing the laser energy typically results in larger maximum diameters, both when the bubble size is measured directly from high-speed imaging and when it is calculated using Eq. 3 (Table 3). The size of individual bubbles deviates from the average value by approximately 17%. This fluctuation can be attributed to the formation of elongated plasma and the generation of smaller bubbles following the collapse of the laser bubble. Occasionally, these smaller bubbles may merge with the subsequent laser bubble, leading to asymmetrical and larger-sized bubble formation.

**Table 3:** Summary of results for the max diameter of the laser-induced bubbles, collapse time of the bubble’s first two oscillations, and the energy partition.

$E_T$ (mJ)	55	74	81	95
Max diameter (Exp.) (mm)	2.9	3.4	3.5	3.6
Max diameter ($R_{c}$) (mm)	3.2	3.6	3.6	3.9
$E_B$ (mJ)	1.3	2.1	2.2	2.5
$E_{s2}$/$E_B$ (%)	31.9	26.7	25.9	43.5
Energy release through $E_B$ and $E_{s1}$ (mJ)	3.2	4.8	5	6.3

The Rayleigh-Plesset equation consistently yields maximum bubble diameters that are on average 5.8% larger compared to the measurements obtained through experimental visualization (Table 3). This might be attributed to the simplifications made within this model.

Table 3 presents the summarized data on the conversion of the total laser energy into mechanical energy during two distinct phases: cavitation bubble formation ($E_{B}$) and shockwave propagation within the medium ($E_{s1}$). Besides plasma generation, $E_{B}$ also serves as a secondary energy source responsible for the generation of radicals within the laser-exposed side of the cavitation box. Both $E_{s1}$ and $E_{B}$ also play roles in the chemical activity occurring within the secondary bubbles side in the cavitation box, with the latter demonstrating a relatively diminished influence. Notably, a relatively higher increase in total laser energy release ($E_{s1}$ + $E_{B}$) is observed at a laser energy of 95 mJ, which explains the higher rate of chemical activity at this laser energy within the secondary bubble’s side (Fig. 12b).

The average energy loss of the laser-induced bubble due to sound emission during its first collapse at the highest value is 43.53% ($E_{s2}$/$E_{B}$). This falls below the results reported in the literature, which average around 73%^41^. A possible explanation for this discrepancy is that the bubble shapes deviate from a perfect sphere shape, especially for the smaller bubbles in our experiment; as a result, the bubble implodes less violently than a spherical collapse, leading to reduced sound emission.

**Figure 8:**
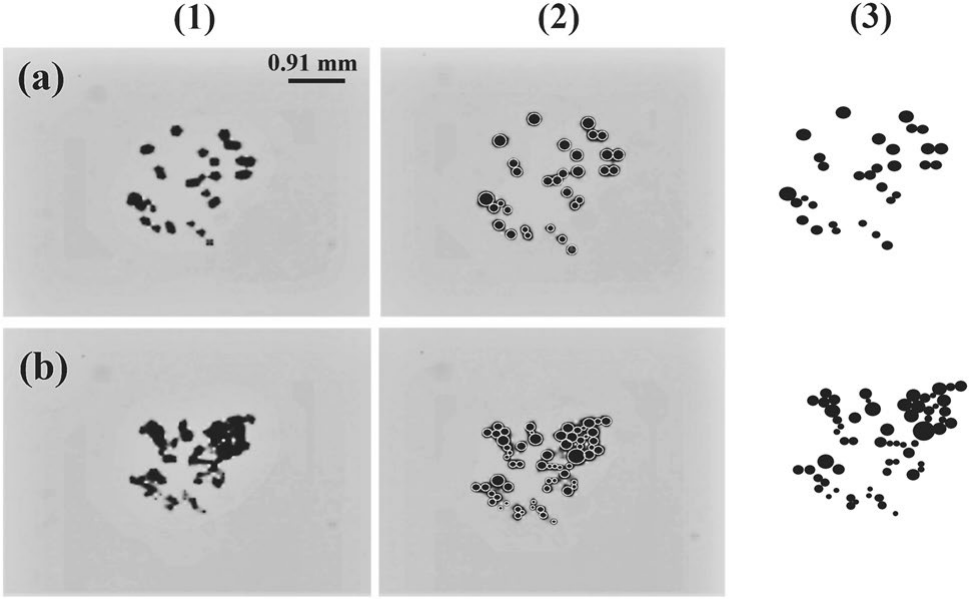
Secondary bubble clusters: (**a**) first set, (**b**) second set. (1) raw images, (2) overlay masks, and (3) enhanced masked images for further analysis at 81 mJ laser pulse energy.

The results from the analysis of secondary bubble clusters reveal the effect of laser pulse energy on the number density and size of these bubbles. The bubble size distribution, the variation of bubble counts, the average bubble diameter, and the average number density of bubbles per pulse are determined at different laser pulse energies. The process of detecting secondary bubbles involves three main steps (Fig. 8): the first is pre-processing, which requires manually defining an overlay mask on each cluster at its largest size (Fig. 8(2)). The mask is based on boundary curvature and aims to estimate the maximum number of bubbles of different sizes that are present in a cluster. The second step focuses on the segmentation of overlapping bubbles using the watershed technique. Finally, the Matlab code detects each bubble and incorporates its size into the computed distribution for each laser pulse (Fig. 8(3)).

**Figure 9:**
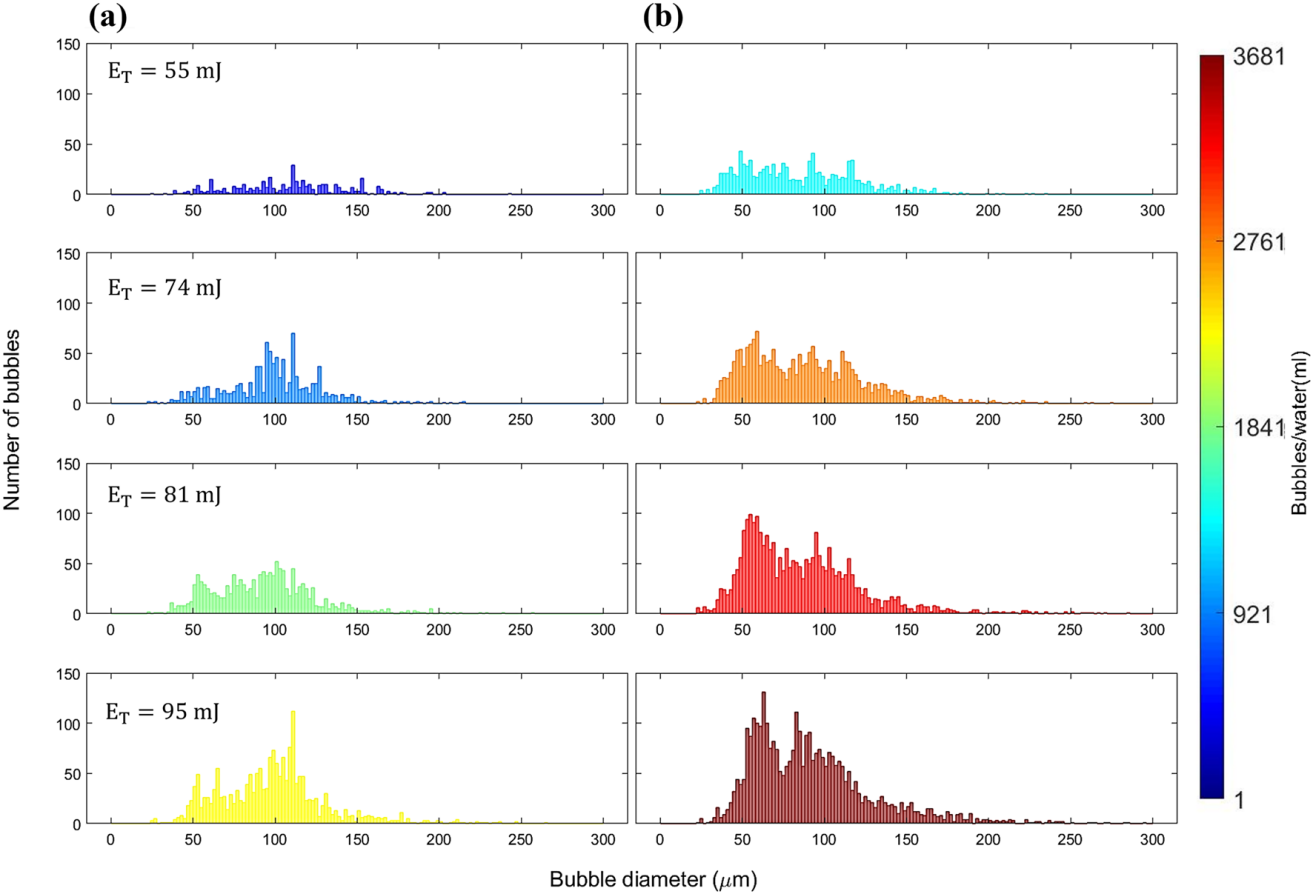
Size distribution of bubble in (**a**) the first set, (**b**) the second set of secondary bubble cluster formation.

Figure 9 illustrates the size distribution of cavitation bubbles in each cluster as a function of laser energy. The vertical axis is the bubble count within each size range. The histogram comprises 150 bins with a width of 2 $\upmu$m. The color in the display represents the bubble number density per pulse, which is calculated as the average number of bubbles produced per pulse divided by the average volume of the cluster. The estimation of the cluster’s volume is based on high-speed imaging. In this process, we determine the smallest circle that completely encircles all the bubbles within a cluster. Subsequently, we compute the volume of a sphere equivalent to this circle, representing the volume of the cluster. In our experiments, the average volume of the clusters is found to be 0.02 ml. It is observed that with an increase in laser energy, the number density of bubbles also increases monotonically.

Initially, the size distribution for both sets displays an unimodal pattern. However, for input energies exceeding approximately $E_{T}$ = 81 mJ, the distribution transitions to a bimodal pattern. In the first set, the dominant mode corresponds to a larger bubble size, while in the second set, the dominant mode is associated with a smaller bubble size. As laser energy increases, the number of bubbles within each mode also increases. The bubble number density within a cluster differs between the two sets, with a higher value observed when the CSW triggers the formation (second set). The observed size distribution can be attributed to a stronger BSW, as indicated by the hydrophone signal. This phenomenon leads to the formation of more spherical and distinct large cavitation bubbles in the first set of bubbles, in contrast to the second set. However, it is worth noting that we also observe large bubbles in the second set, which we believe are primarily merged bubbles within the clusters. This merging is likely a result of the high number of small bubbles in close proximity within the clusters. Alternatively, the collapsing bubbles in the first set may form nanobubbles, which are too small to be seen by the visualization technique due to the limited resolution of the camera. As the CSW travels over the focal point of the reflector, these nanobubbles serve as nuclei and result in the formation of clusters with high bubble density but smaller in size due to the weaker shockwave (Fig. 9b).

To determine the relationship between the size distribution of the bubbles in a cluster and laser energy, we combine the bubbles from both sets and obtain a single size distribution for each laser pulse energy.

**Figure 10:**
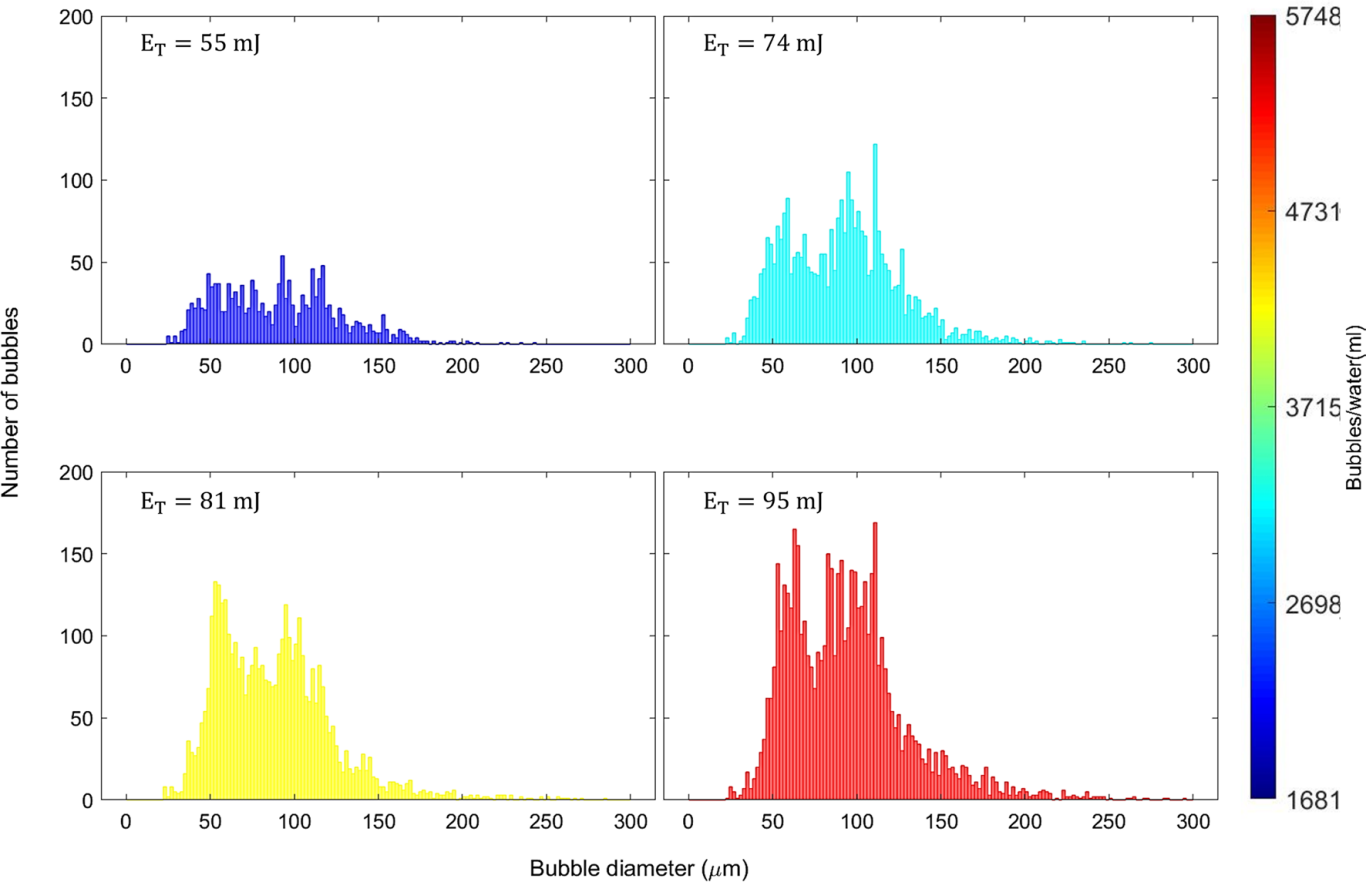
Total size distribution of cavitation bubbles.

Figure 10 presents the size distribution of the total number of bubbles as a function of laser energy. The bubble number density increases monotonically with energy. The bubble size distribution exhibits a bimodal pattern across all energy values, with the dominant mode corresponding to larger bubbles, except at 81 mJ.

**Table 4:** Average size and average bubble number density in every pulse irradiation.

$E_T$ (mJ)	55	74	81	95
Average bubble diameter ($\upmu$m)	90±15	91±12	88±8	95±9
Average bubble number per pulse	33±11	71±19	90±25	120±28
Average bubble number density per pulse (bubbles/ml)	1650	3500	4500	6000

Table 4 shows the average bubble diameter, the average bubble number per pulse, and the average number density of bubbles per pulse increase as the laser pulse energy increases, reaching their maximum values of $95\,{\upmu }\hbox {m}$, 120 and 6000 bubbles/ml, respectively, at a laser energy of 95 mJ. However, a slight decrease in the average bubble diameter is observed at 81 mJ, indicating a higher proportion of smaller bubbles in a cluster in contrast with the other laser energy size distribution. This anomaly can be ascribed to a transient decreasing trend at the 81 mJ energy within the general increasing trend of the $E_{s1}$ to $E_{T}$ ratio as laser energy increases. In general, increasing the laser pulse energy results in stronger shockwaves, which can lead to the formation of clusters with higher bubble number density. However, the likelihood of interactions between the bubbles also increases in these clusters and the precise effect on bubble size in the cluster may vary depending on the degree of energy increase and the strength of the shockwaves.

**Figure 11:**
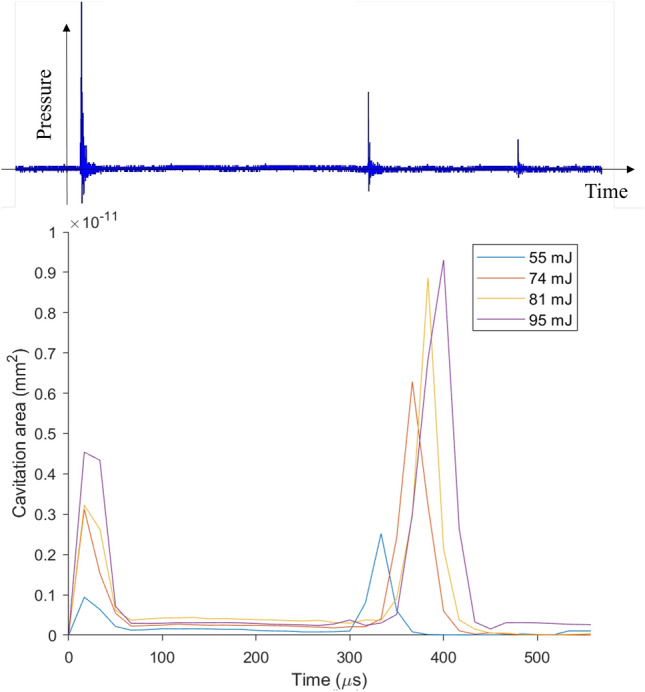
Relationship of generated cavitation area with a time-lapse of laser pulse exposure.

To determine the lifespan and persistence of secondary cavitation bubbles; a characteristic influenced by their maximum size and the intensity of their collapse, we traced the dynamic evolution of their combined projected area within a target region measuring $7.42 \times 3.71~\textrm{mm}^2$. This analysis was performed across different laser energy levels, with the findings illustrated in Fig. 11. The measured area is derived from the count of pixels representing bubbles (depicted in black) within the captured images. The summation of these active pixel areas corresponds to the projected cluster area within the medium. The time is recorded from the moment the primary shockwave (BSW) reaches the focal point until the time the second CSW, based on the collapse time of the laser-induced bubble, travels over the focal point of the reflector (about 557 $\upmu$s). Figure 11 shows the duration and starting time of each set of bubbles. The first set of bubbles form simultaneously and disappear after 50 $\upmu$s, but the starting time of the second set of bubbles varies with the laser energy, with higher energies resulting in longer collapse times and delayed starting times. The second set of bubbles is observed to persist for a longer duration at the focal point as compared to the first set, and its lifetime increases with an increase in the laser pulse energy. A potential explanation for this observation is the higher void fraction in the second bubble cluster, which could extend the overall collapse time^53^. As the cluster grows in size and number density, the bubbles located centrally are subject to delayed collapse. This delay is primarily due to the shielding effect exerted by the peripheral bubbles, effectively protecting the inner bubbles from immediate collapse. In a typical bubble cluster, the collapse process initiates in a layered manner, starting from the outer regions. During this initial phase, the central bubbles maintain a larger volume and are situated in a comparatively low-pressure zone. Subsequently, these outer bubbles create a higher-pressure environment, which then propels the collapse of the inner bubbles^54^. Additionally, the interaction among the bubbles within the cluster leads to a less intense collapse of the second set of bubbles. This reduced intensity allows them to remain and continue oscillating within the cluster.

### Amount of generated ROS

The generation of hydroxyl radicals ($\hbox {OH}^{.}$) is a typical occurrence at the implosion of cavitation bubbles. $\hbox {OH}^{.}$ are highly reactive and oxidant (oxidation potential of 2.81 V)^55,56^. We measure the oxidative reaction of hydroxyl radicals on both sides of the cavitation box using the KI method.

**Figure 12:**
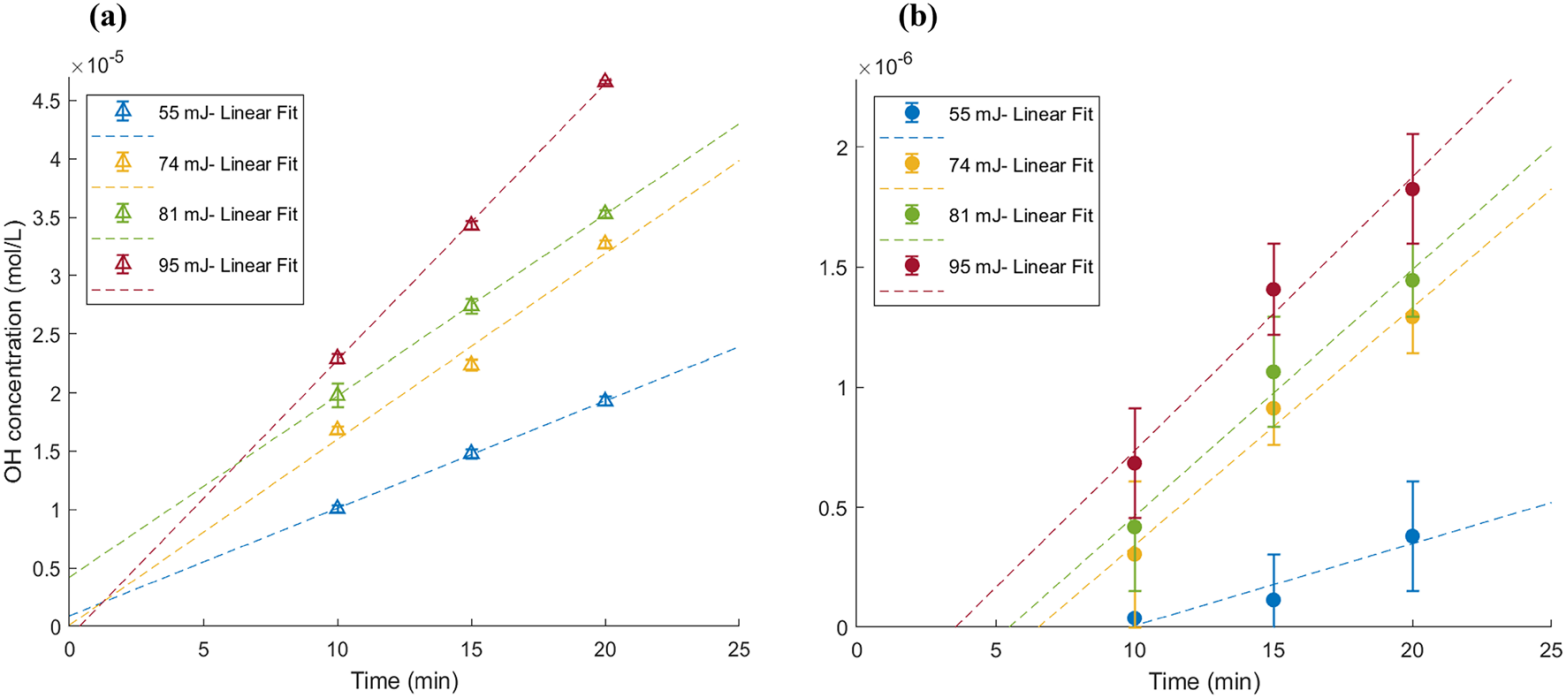
Hydroxyl radicals ($\hbox {OH}^{.}$) concentration at different laser energies and irradiation times, (**a**) laser bubble side, (**b**) secondary bubbles side of the cavitation box, (Linear fit lines are added to each data set to assess their linearity).

Figure 12 displays the $\hbox {OH}^{.}$ concentration on both sides of the cavitation box as a function of irradiation time, with 10 laser pulses per second. The concentration in the solution of the laser side is shown to be significantly higher, at over 20 times that of the secondary bubble side. The notable difference in $\hbox {OH}^{.}$ concentrations shown in Fig. 12 is attributed to the extreme conditions of heat and pressure from the laser-induced plasma itself in addition to bubble collapse, which enhance water molecule dissociation and the generation of $\hbox {OH}^{.}$ on the laser side. While the plasma could theoretically lead to the formation of a large number of radicals, it simultaneously increases the local water temperature. Entezari’s research^57^ indicates that such temperature rise can significantly reduce the sonochemical oxidation of iodine, particularly above 55 ^∘^C where the reaction rate substantially declines. The plasma generated by the laser can cause localized heating. Although the overall bulk temperature of the water consistently stays well below 55 ^∘^C, the region immediately surrounding the plasma can reach much higher temperatures momentarily. This transient increase in temperature of the regions with high concentrations of $\hbox {OH}^{.}$ radicals can lead to a reduction in the sonochemical oxidation of iodine. In our experiments, we obtain a substantially lower concentration of $\hbox {OH}^{.}$ radicals compared to the findings of Peng et al.^31^, who observe $4.8 \times 10^{-5}$ moles of $\hbox {OH}^{.}$ radicals per laser pulse in a bubble with a 1.58 mm radius, generated at a laser energy of 10 mJ. Our experiments, employing a 55 mJ laser pulse, produced bubbles of similar size, potentially a result of the laser beam’s focusing angle within the water medium. We recorded approximately $1.2 \times 10^{-10}$ moles of $\hbox {OH}^{.}$ radicals per pulse in the laser region of the cavitation box, a discrepancy likely stemming from our use of degassed water. This factor reduces oxygen levels in the medium, consequently, the reactions leading to $\hbox {OH}^{.}$ radical formation. The methodology employed for measurement also significantly influences the results. We employed the KI dosimetry technique, which is highly dependent on the concentration of $\hbox {H}_{2}\hbox {O}_{2}$ and its oxidative effects. Additionally, our results are somewhat more aligned with those of Pawlak et al.^36^, who used femtosecond laser pulses in degassed water and reported $1.25 \times 10^{-13}$ $\hbox {OH}^{.}$ radical moles per pulse. Our measured values are higher than theirs, a variance that could be explained by the smaller size of bubbles induced by femtosecond laser pulses and lower laser energy (1 mJ). In such cases, the generation of radicals from bubble collapse is considerably less pronounced. Figure 12 illustrates the effect of the laser pulse energy and the duration of the exposure (number of total bubble collapse) on the formation of $\hbox {OH}^{.}$. Both factors enhance $\hbox {OH}^{.}$ formation on both sides of the cavitation box, with a decrease in the effect of laser energy at 81 mJ, followed by an initial increase. Figure 11 reveals that the total cavitation bubble projected area at 81 mJ in the first cluster is similar to that at 74 mJ; However, the second cluster is closer to the cavitation area observed at 95 mJ. This implies that the first set of bubbles affects the chemical activity more than the second set of bubbles due to its large spherical shape bubbles caused by low bubble-bubble interaction. Conversely, the second set of bubbles is smaller and more closely spaced, likely leading to increased interaction and asymmetrical collapse (lower temperature and pressure at the collapse region) and, subsequently, lower chemical activity.

By degassing the medium before the experiments, we assume that the diffusion of dissolved air into the bubble during expansion is negligible. As a result, the primary reactions taking place within the collapsing bubble, which lead to the generation of $\hbox {OH}^{.}$, are as follows:

(1) $$\hbox {H}_{2}\hbox {O} \rightarrow \hbox {OH}^{.} + \hbox {H}^{.}$$

(2) $$\hbox {H}^{.} + \hbox {H}_{2}\hbox {O} \rightarrow \hbox {OH}^{.} + \hbox {H}_{2}$$

The standard enthalpy change ($\Delta H^\circ$) for a chemical reaction can be determined by calculating the difference between the standard enthalpies of formation for the products and reactants. We obtain the standard enthalpies of the products and reactants from GRI-Mech 3.0^58^. The energy requirements for reactions 1 and 2 are +499.15 kJ/mol and +63.17 kJ/mol, respectively. The combined reaction is $2 \hbox {H}_{2}\hbox {O} \rightarrow 2 \hbox {OH}^{.} + \hbox {H}_{2}$, with a total enthalpy change of +281.16 kJ/mol. This value represents the energy required for the formation of 1 mole of $\hbox {OH}^{.}$ radical in the system.

**Figure 13:**
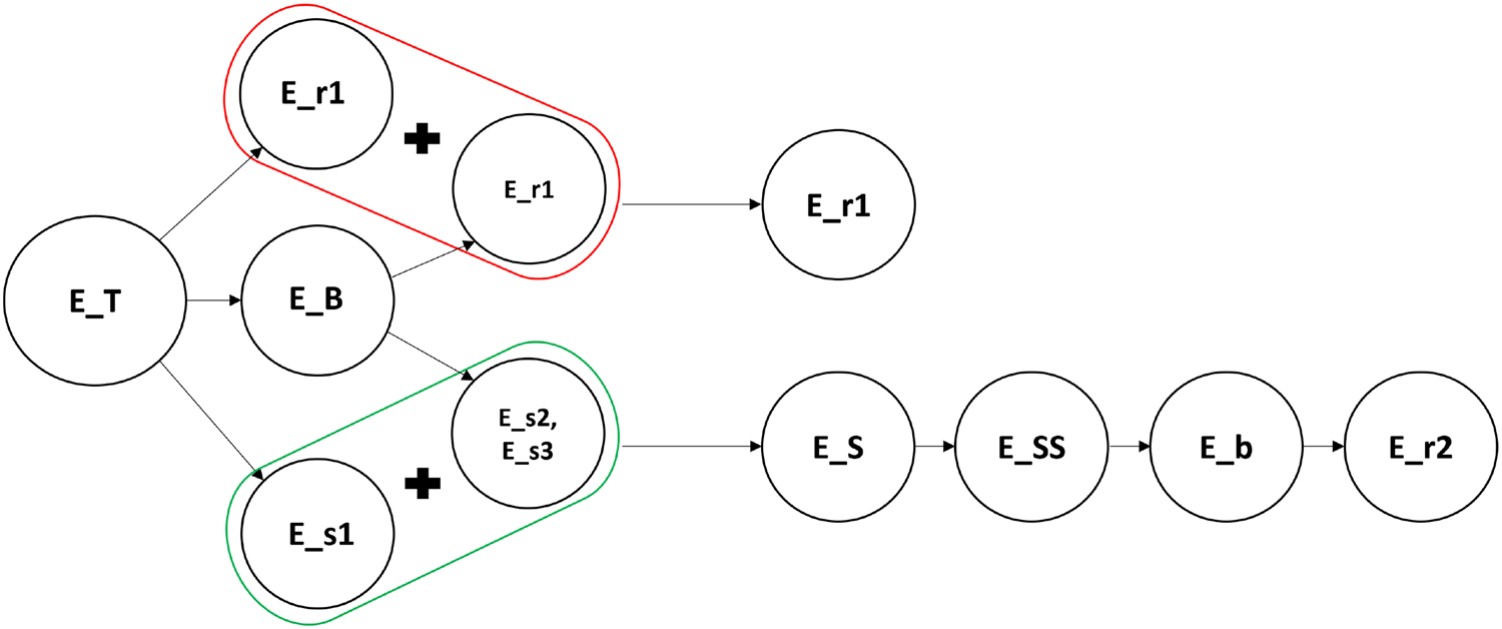
Schematic representation of energy conversion from laser pulse to chemical energy in the cavitation box.

Based on the reaction energy and the concentration of the final product in KI dosimetry, we obtain the energy absorbed through the formation of radicals both on the laser side ($E_{r1}$) and the secondary bubbles side ($E_{r2}$), which is summarized in Table 5. To highlight the different pathways of the energy conversion process, a diagram illustrating the transformation of laser pulse energy into chemical energy on both sides of the cavitation box is presented in Fig. 13.

**Table 5:** Summary of results for the chemical reaction energy.

$E_{T}$(mJ)	55	74	81	95
$E_{r1}$ (${\upmu }$J)	22.6	34.1	38.9	47.3
$E_{r2}$ (${\upmu }$J)	0.2	0.9	1.1	1.5
$E_{r1}$/$E_T$ (%)	0.04	0.05	0.05	0.05
$E_{r2}$/$E_{SS}$ (%)	3.8	14.3	16.5	14.9
$E_{b}$/$E_{SS}$ (%)	45.8	66.5	79.3	87.1

However, in this study, we have not considered the effect of the plasma and of the subsequent ions, radicals, and atoms produced due to its formation that diffuse into the laser-induced bubble during its initial expansion. As a result, the reactions occurring on the laser side of the cavitation box may be more complex than those on the secondary bubble side.

The chemical reaction energy on both sides of the cavitation box increases with the increase in laser energy. Table 5 presents the rates at which source energy is harnessed into chemical reaction energy. For secondary bubbles, it is assumed that their formation energy derives from a fraction of the energy of the shockwave, which is focused by the reflector at each incremental laser energy input, denoted as $E_{SS}$. To quantify this, we measured the pressure amplitude at the location of the reflector by applying the 1/r law to the hydrophone measurements. Subsequently, employing Eq. 1, we determined the shockwave energy at the reflector’s position. It is estimated that the reflector concentrates approximately 4% of this shockwave energy (based on its size and geometry), contributing to the formation of the secondary bubbles. On the laser side of the cavitation box, the source energy is defined by the focused laser energy within the bulk medium ($E_T$).

The efficiency of chemical energy conversion in the cavitation box is notably low on both sides, with the laser-induced side ($E_{r1}/E_T$) being even less efficient than the secondary bubbles side ($E_{r2}/E_{SS}$). A potential explanation for the low efficiency on the laser-induced bubble side is the increase in the medium’s temperature coupled with the shock waves generated by high-energy plasma pulses, leading to the decomposition of $\hbox {H}_{2}\hbox {O}_{2}$ into water ($\hbox {H}_{2}\hbox {O}$) and oxygen ($\hbox {O}_{2}$)^31^. Furthermore, the recombination of radicals before engaging in chemical reactions, and imperfections in the laser beam could also contribute to this observation. Additionally, it’s important to consider that not all parts of the laser beam are effectively contributing to the formation of plasma, which could further account for the observed inefficiency. On the side of the secondary bubbles in the cavitation box, a significant portion of the reflected shockwave energy is dissipated beyond the focus point, failing to contribute to the formation of secondary bubbles or to increase the energy within the cluster. This loss of energy, occurring away from the focal area, is likely the dominant factor to the low energy conversion ratios. The conversion rates on the secondary bubble side of the cavitation box generally show an upward trend with increasing laser energy, yet an anomaly occurs at 95 mJ where a minor reduction in the conversion rate is observed on the secondary bubble side. Here bubbles are the only means to transform the shockwave energy into chemical energy. To understand this transformation, we compute the energy stored in secondary cavitation bubbles using Eq. 4 and the size distribution of the bubbles (Fig. 10). We assess the efficiency of converting focused shockwave energy into stored energy ($E_{b}/E_{SS}$) as presented in Table 5. Interestingly, no decline in this particular conversion rate is observed, suggesting that the decrease at 95 mJ is probably attributable to the altered dynamics of the cavitation bubbles and their less intense collapse.

### Empirical model

We propose an empirical correlation, based on the experimental data of secondary cavitation bubbles, to estimate the amount of energy consumed for the decomposition of water vapor molecules due to the collapse of the secondary bubbles in a solution. The model accounts for the energy stored in the cluster resulting from the formation of cavitation bubbles. Utilizing the Matlab curve fitting toolbox, we estimate the proportion of cluster energy that is converted into chemical energy in the solution, originating from a cluster characterized by a specific energy density.

5 $$\begin{aligned} Cr= 1.324 E_{d}^{3}-7.987 E_{d}^{2}+9.526 E_{d}+22.86 \end{aligned}$$

Where, *Cr* = f ($E_{d}$), $E_{d}$ represents the cluster energy density (J/L), and *Cr* is the conversion rate (%) of the cluster energy to the energy released in the medium following the collapse of the cavitation bubbles and consumed to the decomposition of water vapor molecules, which is obtained based on the analysis of KI dosimetry experiments (mJ).

**Figure 14:**
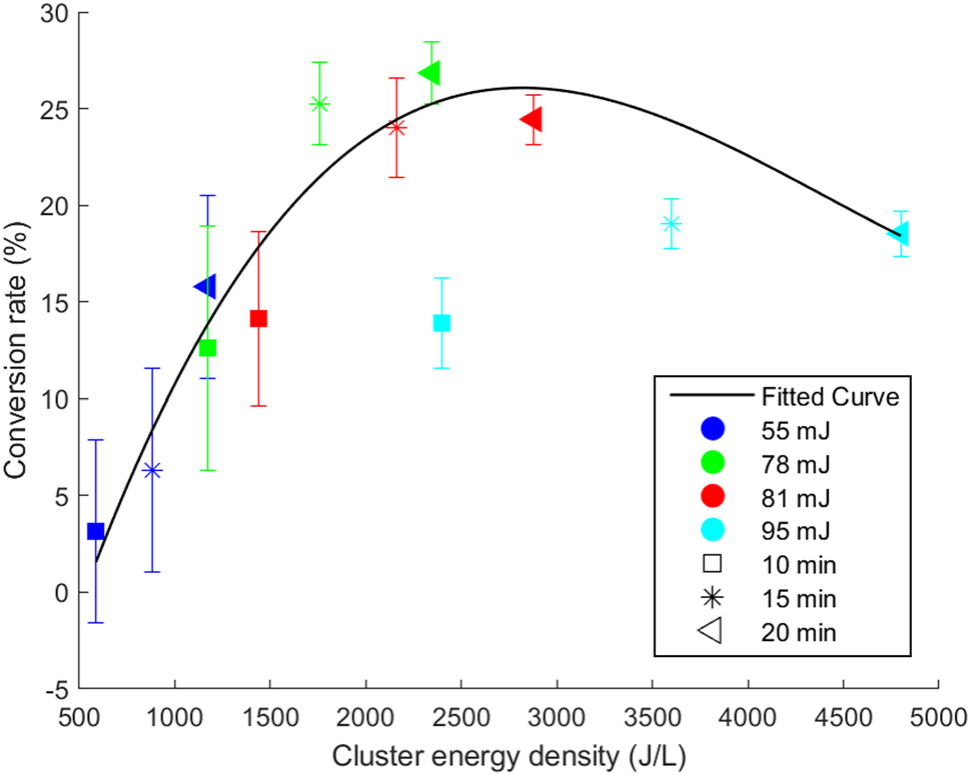
Curve fitting of secondary cavitation bubble dynamics across various laser energies and irradiation durations. RMSE = 2.748, $R^2=0.9253$.

The fitted curve depicts the rate at which cluster energy is transformed into chemical energy, as a function of the cluster’s energy density (Fig. 14). The size and number of bubble collapses play a key role in determining the overall energy and, consequently, the chemical activity of the cluster. Initially, as the cluster energy density increases, there is a corresponding enhancement in the decomposition of water vapor molecules during the collapse of the cavitation bubbles, leading to a higher release of energy. This phenomenon can be attributed to an increase in either the size or number of bubbles within the cluster. This relationship is particularly pronounced at lower cluster densities, where the conversion rate swiftly escalates, demonstrating a robust direct correlation within this domain.

As the energy density is further increased, the conversion rate correspondingly climbs, peaking at an optimal level that signifies the most efficient cluster energy density for energy conversion. Beyond this peak, a further increment in energy density results in a decline in conversion efficiency, as the curve demonstrates a downward trend, indicating a less effective energy conversion rate at higher densities. Several factors contribute to this behavior, including limitations in the availability of reactants and the occurrence of merging and coalescence phenomena within the cluster. The data in Fig. 14 reveals that clusters with approximately equal stored energy densities can exhibit varying conversion rates, which means different chemical energy releases within the medium. Among the three cases shown in Fig. 14 with approximately 2400 J/L cluster energy density, the most favorable conversion rate is achieved when input shockwave energy is relatively low (laser energy of 74 mJ) and the irradiation time is long (20 min). Under these conditions, the bubbles are relatively large but the clusters are not too dense to prevent excessive bubble-bubble interactions that compromise spherical collapse. The clusters characterized at a higher laser energy of 81 mJ, summed up for a period of 15 min to obtain a similar 2400 J/L for the total energy, exhibit somewhat lower conversion efficiency to chemical energy. In contrast, the clusters formed under the highest laser energy of 95 mJ for a 10-minute exposure are associated with the lowest chemical energy, presumably because of the higher bubble number density. For the three data points discussed above, we observed a twofold decrease in chemical energy conversion between the two extreme cases, at approximately equal total cluster energies. For the most favorable case, the average bubble diameter is 91 $\upmu$m and the bubble density is 3500 bubbles/ml, resulting in a dimensionless bubble-to-bubble distance ratio of 8, calculated by dividing the average distance between bubbles by the average radius of the bubbles in a cluster. For the least favorable case, the average bubble diameter is 95 $\upmu$m and the bubble density is 6000 bubbles/ml, resulting in a dimensionless bubble-to-bubble distance ratio of 6.3. As the cluster gets denser, the likelihood of bubble interactions and subsequent bubble merging increases. The coalescence of bubbles results in a redistribution of energy within the cluster, causing a decrease in the overall energy release per bubble, particularly during non-spherical bubble collapses. This observation aligns with the energy dampening effect that becomes apparent when the cluster energy density exceeds the optimum level, which is approximately 2500 J/L based on Fig. 14. This outcome serves as a notable illustration that amplifying the input energy doesn’t necessarily result in enhanced outcomes. This pattern underscores the significance of achieving an optimal balance of bubble number density within a cluster and standoff distance to enhance chemical activity and as a result efficiency of the sonochemical reactions.

As reported by Hussain et al.^59^ the issue they have with scaling up of the sonoreactors is their low sonochemical efficiency, which can be attributed to their very high bubble density. Sonocavitation typically features a bubble density of about $10^4$ bubbles/ml of water, as cited by Fang et al.^60^, which is significantly higher than the 3500 bubbles /ml observed here. However, there is a slight difference between sonocavitation and the secondary bubbles we have here. In sonoreactors, sonocavitation bubbles undergo strong oscillation due to the alternating compression and rarefaction cycles induced by ultrasound. This oscillation gives rise to the secondary Bjerknes force, a phenomenon resulting from the pressure fields generated by the pulsating bubbles. A pulsating bubble emanates a pressure wave through the surrounding fluid, impacting nearby bubbles. The interaction of these pressure waves can cause the bubbles to either coalesce or repel each other. Consequently, the required standoff distance among sonocavitation bubbles to avoid coalescence is determined by the amplitude and frequency of the acoustic field. Nevertheless, it is evident that to enhance sonochemical efficiency, sonoreactor design should pivot towards reducing bubble density and increasing both bubble size and their standoff distance. It has also been observed that the first set of bubble clusters consistently exhibits higher chemical activity compared to the second round, attributable to their larger size and spherical shape. Consequently, it can be concluded that employing pulse mode ultrasound, as opposed to continuous mode, is an effective strategy to reduce the likelihood of forming clusters with a high density of bubbles in close proximity.

## Conclusion

We develop a novel experimental tool to study the chemical effects of cavitation bubble clusters in water using laser pulses. We first determine bubble characteristics and then quantify the fundamental chemical processes within laser-induced and secondary cavitation bubbles. Secondary cavity clusters form near a concave boundary reflecting laser-induced shockwaves and we isolate them from laser-induced bubbles using an aqualene elastomer. Shadow photography reveals bubble cluster structures and behavior, indicating that the breakdown shock wave (BSW) generates the strongest secondary cavitation, followed by the first and second collapse shock waves (CSWs), with the latter having the least impact.

The conversion of shockwave energy to chemical energy is facilitated by the formation of bubble clusters. We investigate the effect of bubble cluster properties on the generation of $\hbox {OH}^{.}$ employing both the KI method and high-speed camera imaging. With the increase of the laser energy, stronger shockwaves are generated, which in turn yield larger bubbles and increase the bubble density within a cluster. These factors positively influence chemical reactions, leading to a significant increase in the generation of $\hbox {OH}^{.}$. The concentration of these radicals has been observed to reach values as high as $1.82 \times 10^{-6}$ mol/L in our experiments.

Hydrophone measurements reveal that in our favorable case, 16.5% of the focused shockwave energy contributes to the decomposition of water vapor molecules and the formation of hydroxyl radicals. However, as we increase the shockwave energy, the conversion rate decreases to 14.9%, which is attributed to the behavior and intensity of the cavitation bubbles’ collapse within a cluster; Since the transmission of shockwave energy into the cluster consistently demonstrates an upward trend. The empirical correlation derived from the experimental data of secondary bubbles enables the prediction of a bubble cluster’s potential in converting mechanical energy into chemical energy required for reactions. An increase in cluster stored energy initially leads to an increase in chemical energy. However, the optimum conversion rate is obtained at a cluster energy density of approximately 2500 J/L (cluster with an average bubble diameter of 91 $\upmu$m, bubble density of 3500 bubbles/ml, and dimensionless bubble-to-bubble distance ratio of 8), which highlights the role of bubble interaction, sphericity, and the availability of reactants in the medium.

## Data Availability

The datasets used and/or analyzed during the current study are available from the corresponding author upon reasonable request.
